# Transcranial direct electrical stimulation for hand function in a stroke patient with severe upper limb paralysis due to lenticulostriate artery occlusion: a case report

**DOI:** 10.1186/s13256-021-03137-1

**Published:** 2021-12-13

**Authors:** Koichiro Hirayama, Takeshi Fuchigami, Shu Morioka

**Affiliations:** 1 Department of Rehabilitation, Eishinkai Kishiwada Rehabilitation Hospital, 8-10, Kanmatsucho, Kishiwada, Osaka 596-0827 Japan; 2 Graduate School of Comprehensive Rehabilitation, Osaka Prefecture University, Osaka, Japan; 3 Stroke Rehabilitation Research Laboratory, Eishinkai Kishiwada Rehabilitation Hospital, Osaka, Japan; 4 Neurorehabilitation Research Center, Kio University, Nara, Japan

**Keywords:** Stroke, Hands, Transcranial direct current stimulation, Case report

## Abstract

**Background:**

Transcranial direct current stimulation, a therapeutic modality to modulate the excitability of injured and uninjured brain hemispheres in stroke patients, is expected to be effective in treating upper limb paralysis. We describe the use of transcranial direct current stimulation to improve the function and frequency of use of the paralyzed hand of a patient with lenticulostriate artery occlusion.

**Case presentation:**

A Japanese man in his fifties developed a left internal hindfoot perforator branch infarction owing to lenticulostriate artery occlusion, and presented with severe right upper and lower limb paralysis. Multiple interventions for the paralyzed hand, primarily robot therapy, did not noticeably change his hand function or frequency of use in daily life. Therefore, transcranial direct current stimulation was used in combination with upper limb functional exercises for 20 minutes a day, five times a week, for 6 weeks. Consequently, scores for the hand items of the Fugl–Meyer Assessment of the upper extremities improved, and pain and subluxation around the shoulder joint were reduced. Furthermore, the frequency of use and the quality of movement of the paralyzed hand were improved.

**Conclusions:**

Upper limb functional training and transcranial direct current stimulation improved the function and frequency of use of the paralyzed hand in a stroke patient with severe upper limb paralysis, suggesting that this combined intervention could effectively improve hand function in patients with severe upper limb paralysis.

## Background

Approximately 80% of stroke patients have upper limb paralysis [1], and a severely paralyzed upper limb is unlikely to recover its function with conventional therapy [2]. In stroke patients, the decreased activity of the injured hemisphere and its inhibition by the uninjured hemisphere may interfere with the improvement of upper limb paralysis, which is more pronounced in cases with a greater motor impairment [3]. The reduced function and frequency of use of the paralyzed hand may decrease quality of life by limiting the activities of daily living (ADL), leading to the loss of social roles [4]. Therefore, effective treatments for severe upper limb paralysis are required.

Transcranial direct current stimulation (tDCS), a noninvasive brain stimulation technique, is attracting attention as a treatment for stroke. tDCS involves applying a weak direct current (1–2 mA) to the scalp of a stroke patient. It improves function by modulating the interhemispheric inhibition between the injured and uninjured hemispheres.

Studies investigating the effects of tDCS on brain activity have reported that anodal and cathodal tDCS stimulation increases and decreases cortical excitability, respectively [6, 7]. Furthermore, tDCS improves upper limb function [8] and motor learning [5] in stroke patients.

We report a patient with severe upper limb paralysis whose hand function and frequency of use did not change despite complex interventions (primarily robot therapy) and received tDCS at our rehabilitation hospital. We expected that (1) tDCS would effectively promote the excitability of the injured hemisphere’s primary motor cortex and inhibit the excitability of the uninjured hemisphere, and (2) tDCS combined with upper limb functional training would effectively prevent the decline in function and frequency of use from severe motor paralysis.

## Case presentation

**Fig. 1:**
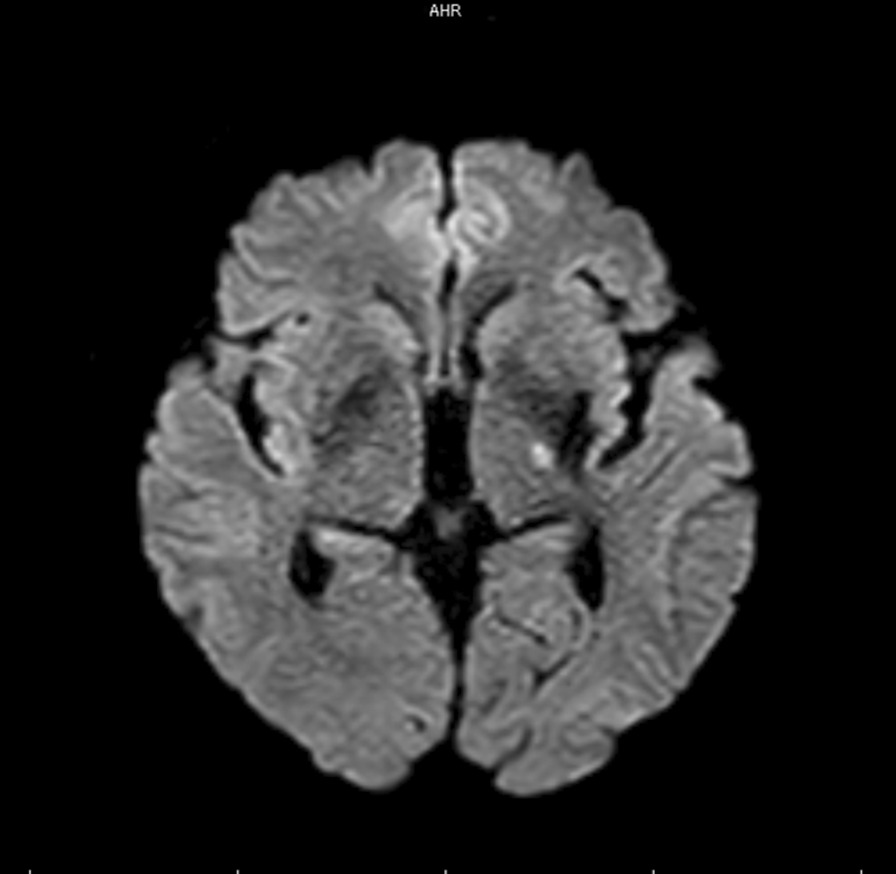
Head CT immediately after onset. The diffusion-weight images of the head showing a lesion of the left posterior limb of the internal capsule reported as a left entropion perforator branch infarction.

A right-handed Japanese man in his fifties was taken to hospital in an ambulance after experiencing numbness in his right upper extremity and difficulty in standing up. A magnetic resonance imaging (MRI) scan was performed immediately after the onset of the symptoms, and the diffusion-weighted images showed a left internal hindfoot perforating branch infarction due to lenticulostriate artery (LSA) occlusion (Fig. 1). The patient had a history of hypertension but no other medical conditions. After receiving treatment at an acute care hospital, the patient was admitted to our rehabilitation hospital on day 15 after the onset of the symptoms. His Fugl–Meyer Assessment score for the upper extremities (FMA–UE) was 4/66 (shoulder/elbow/forearm 4/66 points, hand joint 0/10 points, fingers 0/14 points, coordination/speed 0/6 points); the score for the lower extremities was 7/34 points. He had severe upper and lower limb paralysis, decreased muscle tone around the shoulder joint (Modified Ashworth Scale score 0), subluxation in the scapulohumeral joint (2.0 cm between the acromion and greater tubercle of the humerus), and pain around the shoulder joint (numeric rating scale [NRS] 5). The patient’s Motor Activity Log (MAL) scores for both the amount of use (AOU) and quality of movement (QOM) were 0, and there were no daily situations in which the paralyzed hand was used. His cognitive function was 29/30 on the Mini-Mental State Test, and he had no significant higher brain dysfunction. He required assistance for all daily activities (Functional Independence Measure of Motor subscale 52 points) and moved around in a wheelchair with assistance. Based on the above evaluation, the rehabilitation of the patient included ADL practice, robotic therapy to improve voluntary control of the paralyzed hand’s proximal joints, and bilateral hand movement practice (wiping and sanding) involving the healthy upper limb, and active touch tasks with blocks to improve hand function.

As a result of the intervention, the patient’s FMA–UE score on day 100 was 19 points (shoulder/elbow/forearm 18; manual joint 0; hand 1; coordination/speed 0), but the subluxation remained (1.0 cm between the acromion and greater tubercle of the humerus). In the FMA–UE for hand items, only a few flexor joint movements appeared, and the MAL was unchanged from the time of admission. Therefore, tDCS was added to the intensive training for hand function.

### Intervention

We used a DC stimulator (NeuroConn, Germany) as the tDCS device, and tDCS was conducted via surface sponge electrodes measuring 5 × 7 cm (35 cm^2^). The anode was placed directly above the primary motor cortex of the injured hemisphere (C3 in the 10–20 system), and the cathode was placed directly above the primary motor cortex of the uninjured hemisphere (C4). Each electrode was immersed in water and fixed with a band. The intensity of the stimulation was 2.0 mA, and each session lasted 20 minutes. tDCS was performed five times a week for 6 weeks. These parameters have been previously reported safe and sufficient to produce motor evoked potentials [9, 10].

**Fig. 2:**
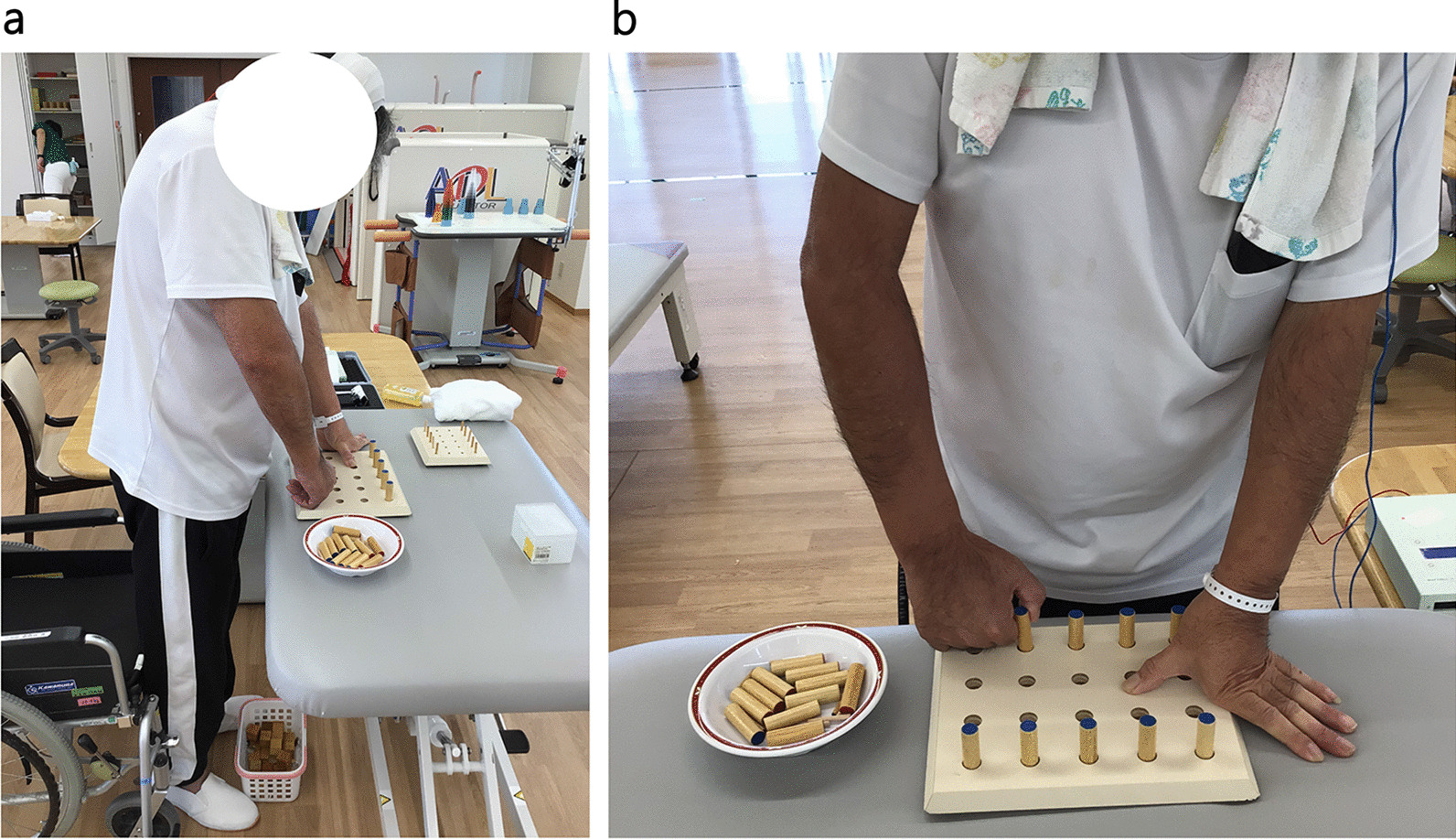
Upper limb functional exercises performed in combination with tDCS. **a** Upper extremity function training involved task-oriented exercises that combined joint movements such as shoulder flexion, elbow extension, and forearm rotation. **b** Step-by-step practice using objects such as pegs and blocks was conducted as hand function improved.

During each 20-minute session of tDCS, upper extremity functional training was conducted (see Fig. 2). The ADL practice included wheelchair operation, toilet operation, and dressing practice according to the patient’s level of independence in ADL.

### Measurements

The upper limb paralysis after stroke was assessed using the FMA-UE [11]. The frequency of use of the paralyzed hand was evaluated by calculating the MAL scores for AOU (MAL-A) and QOM (MAL-Q) [12]. Both FMA and MAL have been translated into Japanese, and the reliability and validity of the assessment methods have been verified. For the subluxation in the scapulohumeral joint, the distance from the acromion to the greater tubercle of the humerus was measured (in cm) and recorded [13]. The degree of pain was assessed using NRS [14]. These evaluations were conducted three times: immediately before, 3 weeks after, and after the tDCS intervention.

**Fig. 3:**
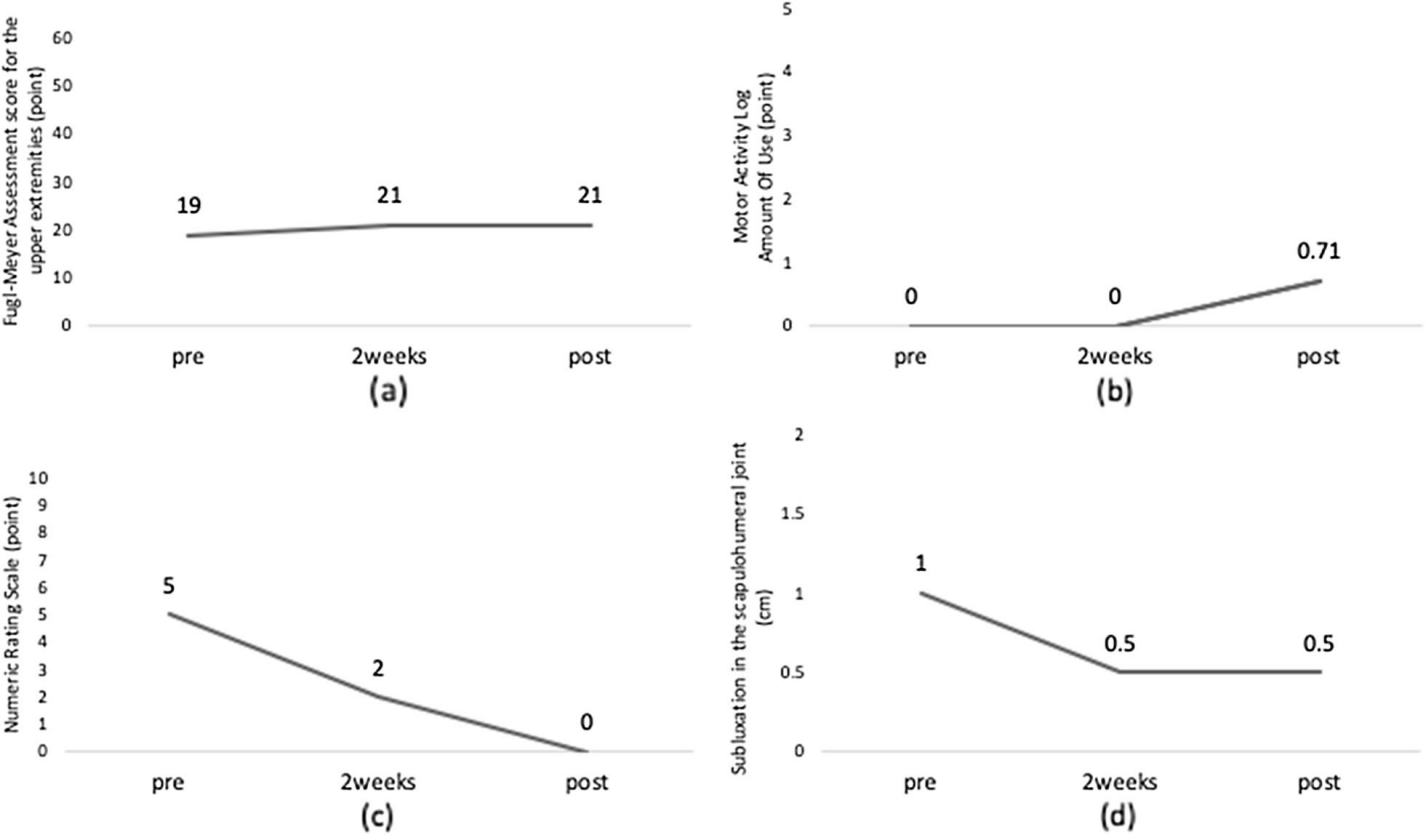
Changes in each upper extremity functional assessment: FMA, MAL, and NRS scores, and the distance from the acromion to the greater tubercle of the humerus pre-assessment, 2 weeks after, and post-assessments **a** The patient was able to flex and extend the hand voluntarily, and the hand items of the FMA–UE scored 2 points. **b** The frequency of use of the paralyzed hand improved by 0.71 points using the motor activity log for amount of use. **c**, **d** The subluxation (subluxation in the scapulohumeral joint 0.5 cm) and pain in the scapulohumeral joint (NRS 0) were no longer symptomatic.

Upper limb function improved following the intervention (see Fig. 3). The patient was able to flex and extend his fingers and was able to grasp and move objects during task-oriented exercises and ADL. In addition, the patient was able to perform ADLs independently using a wheelchair.

## Discussion and conclusions

In this case, we found that tDCS combined with upper limb functional training improved the function and frequency of use of the paralyzed hand of a patient with upper limb paralysis from left medial hindfoot perforator branch infarction caused by LSA occlusion.

A previous study reported that tDCS was effective in regulating cortical excitability and motor activity in stroke patients [15, 16]. In addition, tDCS effectively improved upper limb function based on FMA–UE and Box and Block Test [17].

The effect of tDCS varies depending on the degree of functional impairment at baseline, lesion size, and the difficulty of the task combined with tDCS [18]. Patients with greater disability and lower brain survival may not maximize the effect of tDCS. The case that we reported had small lesions after the LSA infarction, and the tasks in the upper extremity functional exercises that were combined with tDCS were adjusted to match the recovery of hand function. Therefore, the patient may have received the full effect of tDCS, and the function of the paralyzed hand showed improvement. Cho *et al.* reported a greater effect of tDCS on hand function in stroke patients with residual corticospinal tracts [19]. Therefore, the corticospinal tracts may have remained in our patient after the LSA infarction, and tDCS affected the excitability of the injured hemisphere, mainly the corticospinal tracts, leading to the improvement of hand function.

We observed an improvement in frequency of use of the paralyzed hand, which is associated with hand function [20]; therefore, the improvement in the function of the paralyzed hand may have improved the frequency of use.

In this report, tDCS was combined with task-oriented practice, which has been found effective in the recovery of post-stroke upper limb paralysis. Morris *et al.* suggest the ability to perform voluntary extension movements of the fingers as a criterion for introducing task-oriented practice in constraint-induced movement therapy [21]. We suggest that the complex intervention using task-oriented practice in conjunction with the improvement of hand function by tDCS could be useful in patients with severe upper limb paralysis.

The adjustment of activity in the injured and uninjured hemispheres and the increase in excitability of the corticospinal tracts discussed in our report need to be further evaluated using diffusion-weighted tensor imaging (DTI) [22] and functional MRI [23]. Our patient’s intervention included robotic therapy, in addition to the combined treatment of tDCS and task-oriented practice. Therefore, it is difficult to describe the effect of tDCS alone. However, the function and frequency of use of the paralyzed hand did not change despite the combined interventions. This suggests that tDCS may have contributed to the improvement of hand function and the excitability of the corticospinal tract, because hand function is highly dependent on the corticospinal tract.

In conclusion, this case report suggests that combined intervention using tDCS and upper limb functional training improved the function and frequency of use of a paralyzed hand. Therefore, the use of tDCS along with the current approach of upper limb functional training could effectively improve hand function in patients with severe upper limb paralysis and may be a useful intervention strategy.

## Data Availability

All data used were obtained from the patient's medical records in our hospital archives.
